# Robotic Automation of In Vivo Two-Photon Targeted Whole-Cell Patch-Clamp Electrophysiology

**DOI:** 10.1016/j.neuron.2017.08.018

**Published:** 2017-08-30

**Authors:** Luca A. Annecchino, Alexander R. Morris, Caroline S. Copeland, Oshiorenoya E. Agabi, Paul Chadderton, Simon R. Schultz

**Affiliations:** 1 Department of Bioengineering and Centre for Neurotechnology, Imperial College London, London SW7 2AZ, UK

**Keywords:** automated neurophysiology, patch clamp, multiphoton microscopy, robotic automation

## Abstract

Whole-cell patch-clamp electrophysiological recording is a powerful technique for studying cellular function. While in vivo patch-clamp recording has recently benefited from automation, it is normally performed “blind,” meaning that throughput for sampling some genetically or morphologically defined cell types is unacceptably low. One solution to this problem is to use two-photon microscopy to target fluorescently labeled neurons. Combining this with robotic automation is difficult, however, as micropipette penetration induces tissue deformation, moving target cells from their initial location. Here we describe a platform for automated two-photon targeted patch-clamp recording, which solves this problem by making use of a closed loop visual servo algorithm. Our system keeps the target cell in focus while iteratively adjusting the pipette approach trajectory to compensate for tissue motion. We demonstrate platform validation with patch-clamp recordings from a variety of cells in the mouse neocortex and cerebellum.

## Highlights

• A system for robotically targeted neurophysiological recording is demonstrated
• The platform keeps the target in focus and corrects the electrode trajectory
• The system compensates for micropipette-induced tissue deformations
• Targeted recordings were obtained from several fluorescently labeled cells in vivo

## Teaser

Annecchino et al. describe a platform for robotic two-photon targeted patch-clamp recording and demonstrate its functionality by recording from a variety of fluorescently targeted cell types in mouse neocortex and cerebellum.

## Introduction

The patch-clamp technique is an electrophysiological method that allows the recording of macroscopic whole-cell or microscopic single-channel currents in single cells using glass micropipettes filled with an electrolytic solution (Hamill et al., 1981). Whole-cell recording (WCR) provides excellent mechanical stability and current resolution in comparison to other electrophysiological recording paradigms, and is the “gold standard” for high-fidelity analysis of ion channel biophysics and the electrical activity of excitable cells (Sakmann and Neher, 1995, DeWeese, 2007). WCR enables integrative analysis of the molecular, synaptic, and electrophysiological properties of neurons in vitro (Blanton et al., 1989, Edwards et al., 1989, Stuart et al., 1993) and in vivo (Pei et al., 1991, Ferster and Jagadeesh, 1992), including unanesthetized preparations (Covey et al., 1996, Margrie et al., 2002), and can also be used for gene manipulation by the introduction of plasmid DNA into single cells in vivo (Rancz et al., 2011).

When combined with two-photon laser scanning microscopy (2PLSM) (Denk et al., 1990), WCR can be optically targeted to specific cellular structures via synthetic dyes, the use of transgenic animals expressing fluorescent proteins in specific cell types (Margrie et al., 2003, Komai et al., 2006), viral vectors designed to drive expression via cell-specific promoters (Dittgen et al., 2004), or “shadowpatching” (Kitamura et al., 2008). By adaptively planning and executing image-guided pipette motion in the intact brain, it is possible to obtain recordings from specific cells, cell classes, or cell compartments (e.g., dendrites) based on morphological, genetic, or functional signatures. However, the rheological properties of brain tissue make precise, visually guided control of pipette motion challenging. The insertion of a pipette into soft tissue causes viscoelastic morphological deformation, inducing movement of the target. Persistent target position monitoring by an autonomous computer vision system can provide closed-loop visual servoing for robotic micropipette control, allowing automation of the targeting, engagement, and patch-clamping of a fluorescently labeled cell.

Automatic patch-clamp technologies have been used for some years in cell culture paradigms (Fertig et al., 2002), and recently, considerable levels of automation have been achieved in “blind” whole-cell patch-clamp recording (Kodandaramaiah et al., 2012) and image-guided automatic pipette positioning for manual patch clamp in vivo (Long et al., 2015). However, a robotic platform capable of achieving visually targeted electrophysiological recording in vivo, by automatically performing all steps involved in a visually targeted whole-cell patch-clamp experiment, has not until now been demonstrated.

## Results

### Platform and Process Overview

**Figure 1:**
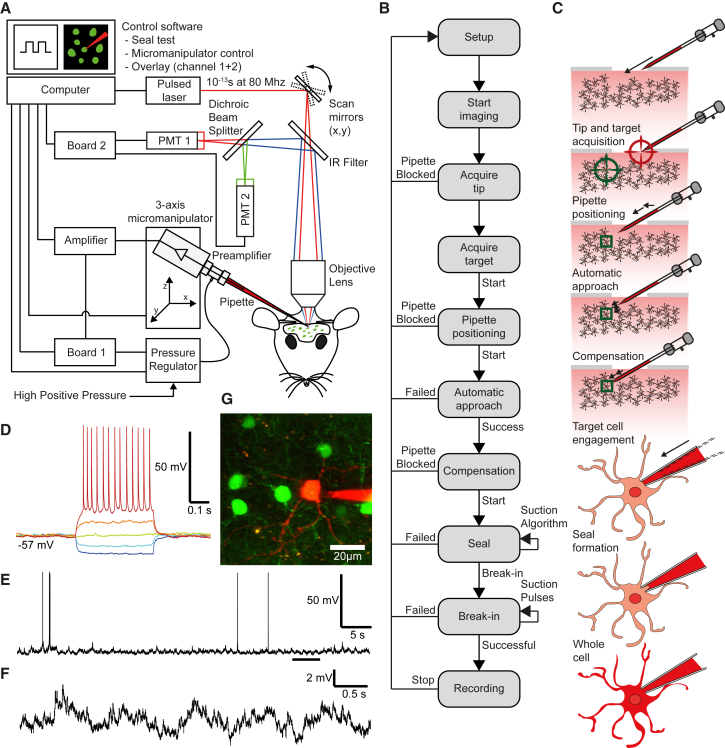
Automated Two-Photon Guided Whole-Cell Recording In Vivo (A) Schematic of the apparatus, which consists of a conventional commercial two-photon microscope, a mode-locked Ti-Sapphire laser, a patch setup equipped with programmable three-axis micromanipulator, a signal amplifier, an analog to digital converter board, a computer, and a custom-made electro-pneumatic actuator for controlling micropipette internal pressure. (B) Block diagram of the two-photon guided robotic procedure. (C) Stages of the visually guided procedure: setup and pipette placement, tip and target coordinate acquisition, pipette positioning, automatic approach, position compensation, target cell engagement, seal formation, and break-in followed by whole-cell configuration. (D–F) Current-clamp traces (D) during current injection (400 ms-long pulses from −100 to +100 pA in 50 pA steps) and (E and F) at rest for a robotically patched gfp-positive neuron in the neocortex of a GAD67-gfp mouse (F is a zoomed-in detail of the underscored section of the trace in E). (G) Two-photon image of the patched neuron and the electrode.

Here we describe a platform capable of performing both robotic “blind” and two-photon guided WCR in vivo (Figure 1A). Our platform integrates a commercial two-photon microscope with a patch-clamp rig and a custom-developed pressure regulator system controlled by a microcontroller running a proportional-integral-derivative (PID) controller algorithm. Custom-developed software written in Labview (National Instruments) controls the whole platform and implements the WCR algorithm (Figure 1B). One difference between our platform and earlier automated blind patch systems (Kodandaramaiah et al., 2012) is the use of closed as opposed to open loop control of pipette internal pressure. As well as allowing a continuous range of pressure values to be selected, and ensuring that the system is robust against fluctuations in input pressure, this is particularly useful for implementing more refined strategies for manipulating pipette internal pressure, mimicking the suction manoeuvers of experienced patch-clamp operators (for instance, the gradual application of suction during seal formation).

The algorithm operates in voltage clamp and automatically performs the principal steps involved in a manually targeted WCR experiment (Figure 1C). After filling a pipette with intracellular solution augmented with a dye such as Alexa 594 and loading it into the pipette holder, the internal pressure is automatically set to a high positive value (10–50 kPa, i.e., 100–500 mBar) by the pressure regulator. A high internal pressure in this initial phase minimizes pipette blockage rate. The pipette is manually positioned 50–100 μm above the exposed pial surface at the center of the field of view for acquisition. The dye-filled pipette tip and fluorescent cells are visualized using multiphoton imaging. Images are continuously acquired while the computer vision system identifies visible objects (cells) in the field of view. Absence of tip blockage is confirmed by a low-impedance readout and a small amount of fluorescent dye ejected from the tip when pressure is applied. The user then focuses on a potential target cell and, if not already acquired, selects it on the screen and saves its 3D location. The system calculates the path of the pipette to the target, aligns its position to the entry trajectory, and rapidly drives it to a point 200–300 μm from the target location.

After pipette positioning, the pipette impedance is checked. If the pipette is not clogged, then automatic approach is initiated. The system automatically guides the pipette toward the target cell while continuously adjusting the trajectory to compensate for target movement throughout the approach process. When the tip is in the vicinity of the target cell (within 20 μm), the internal pressure is reduced to approximately 5–10 kPa. This process ends when the pipette tip reaches the coordinates of the target cell. If the tip is not already in contact with the membrane at the end of the automatic approach, a small manual adjustment (<8 μm depending on the particular manipulator used) of the pipette position can be applied to compensate for any positioning inaccuracy. When target engagement is confirmed by an increase in pipette impedance, the automatic sealing process is initiated.

**Figure 2:**
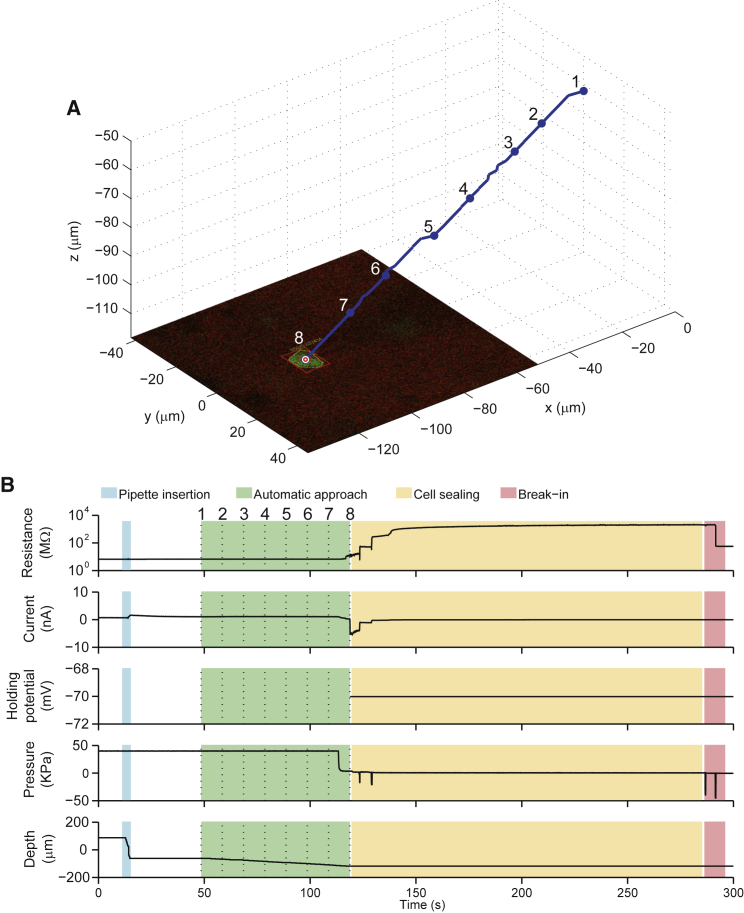
Pipette Approach Trajectory and State during a Typical Robotic Two-Photon Targeted Patching Process (A) Automatic navigation of the pipette toward the target cell, with real-time feedback control of trajectory enabled. (B) Time course of pipette resistance, current, holding potential, internal pressure, and depth during the patching procedure (stages color coded; numeric labels correspond to points on the approach trajectory in A).

In order to facilitate seal formation, the internal pressure is released and a holding voltage (−70 mV) superimposed on the test signal. This results in hyperpolarization (with step or ramp variations) of the pipette and has been shown to help with seal formation (Margrie et al., 2002, DeWeese, 2007). Although sometimes these two manoeuvers are sufficient for a tight seal, in other cases the application of negative differential pressure is necessary, for which we use the closed loop pressure regulator system. A seal is obtained when the pipette resistance exceeds a user-defined threshold (1–1.5 GΩ). If seal formation is unsuccessful, repeated attempts to record from the same cell are possible after replacing the pipette. Cell membrane rupture and access to the cell interior are attained by automatically applying a series of suction pulses. A sharp and rapid decrease of the pipette impedance at this stage indicates successful break-in and attainment of a whole-cell configuration (Margrie et al., 2002, DeWeese, 2007, Kodandaramaiah et al., 2012). A successful trial is confirmed (1) electrophysiologically, by verifying properties such as membrane potential, firing pattern, and access resistance (Figures 1D–1F), and (2) optically, by observing the gradual filling of the target cell with fluorescent internal solution (Figure 1G).

Fine robotic control of pipette position for automatic navigation and target engagement relies on the real-time analysis of the light signature of the fluorescent targets in the intact brain. The insertion of a pipette into soft tissue causes viscoelastic morphological deformation that, in turn, induces target migration away from its initial position. The pattern of motion of objects and features observed results from the relative motion between the scene and the observer (the scope objective). During the target engagement procedure, the current position of the target (i.e., a cell) needs to be continuously reassessed and the approach trajectory of the pipette re-adjusted. In the following sections, we describe how this is achieved.

### Quantitative Analysis of Two-Photon Images

Real-time image segmentation is the first step in this process. Two-photon images are acquired and streamed in real time to a dedicated custom software module responsible for the on-line processing of the incoming data frames. Quantitative information, including contour, area, center of mass, bounding rectangle, and contrast level of individual visible objects in each frame, is calculated. These values are used to track the movement of the fluorescent targets in space and time. Tracking information is then used to adjust the trajectory and dynamics of pipette navigation toward the optical target.

The data frames broadcast by the two-photon microscope system consist of a stream of 16-bit RGB images. On each image in the stream the following operations are performed: channel separation, Otsu thresholding (Otsu, 1979) with background correction, hole filling, erosion filtering, and particle analysis. Image analysis is performed on either a single channel or a combination of red and green channels, after the different layers in the frame have been separated (see Figures S1A–S1E for an example). Otsu interclass variance thresholding with background correction operation allows binary segmentation of the image while performing background correction to eliminate non-uniform lighting effects (Gonzalez and Woods, 2002). This operation is used to identify bright regions corresponding to significant cellular structures for morphological analysis (Figure S1B). Particle analysis performed on the thresholded segments then provides geometrical information that includes the contour, area, bounding rectangle, and center of mass coordinates (Gonzalez and Woods, 2002). Individually labeled segments are used to define pixel masks and probe pixel intensity values in specific regions of the original image (Figures S1C and S1D), allowing calculation of the contrast-focus score (CFS) for each salient object in the image (Figure S1E). The CFS for object *i* is calculated as

(Equation 1) $${\text{CFS}}_{i}=\frac{{\langle I\rangle }_{\text{internal},i}-{\langle I\rangle }_{\text{external},i}}{{\langle I\rangle }_{\text{internal},i}},$$

where ${\langle .\rangle }_{x}$ is the image average operator, over the pixels belonging to the region bounded by contour *x*, with the internal and external boundaries differing due to the erosion filter. This is calculated for each object in all the planes of the original RGB image.

### Tracking and Autofocusing in Two-Photon Time-Lapse Image Streams In Vivo

Target migration is detected by analyzing the spatiotemporal variation of the target light signature at different focal planes. Precise object localization and accurate centroid estimation are prerequisite for reliable determination of motion, prevention of false positives, and reduction of noise-related ambiguities. However, depending on the velocity and acceleration of the optical targets in the visual scene, disruptions in continuity of optic flow may occur. This translates into fluidity degradation of motion appearance (abrupt motion) resulting from insufficient acquisition rate, and poses motion detection difficulties that conventional tracking methods (Deutscher et al., 2000, Isard and Blake, 1998) struggle to handle (Li et al., 2007). Enlarging the search space is computationally difficult, and generally incapable of identifying targets through frames due to loss of image context, increased background clutter, or target migration outside the field of view. In addition, objects whose depth from the focal plane varies in excess of a few micrometers suddenly become severely defocused, and their light signature no longer clearly detectable. For a fixed focal length, dynamic repositioning of the objective is necessary to maintain acceptable visibility of targets migrating along a trajectory not completely enclosed by a single field of view.

Focus variation provides a visual indication that an object is migrating axially (away from the focal plane), and at the same time is a sensitive parameter for regulating object visibility during tracking. The superior optical sectioning capability of two-photon excitation is beneficial in increasing axial resolution but translates to small depth of field, which can be problematic for online 3D target tracking. To preserve object correspondence between image frames and circumvent the intrinsic constraints of a small depth of field and low signal-to-noise ratio, object detection should be achieved before the autofocusing step. The contour and center of mass of the target object can then be used, respectively, as a region of interest for an ad hoc autofocus algorithm and as the initial location of the object in the solution of the inter-frame correspondence problem (Manzo and Garcia-Parajo, 2015). The main goal of inter-frame object correspondence, in this case, is not to reconstruct the object motion track on the basis of noisy measurements, but to detect the exact position of the target object while automatically keeping it in focus during electrode approach. Contrast-based autofocus is achieved by “swiping” the focal plane of the objective over the region of interest and detecting the optical plane along the z axis that maximizes the CFS. We take the best focal distance as the maximum of a Gaussian function fitted to the focus scores (Figures S1F–S1H).

### Automatic Approach to the Target

The rate of viscoelastic deformation of the tissue depends on the dynamics of electrode insertion. By limiting the electrode insertion speed, we limit the distance the target moves between frames, keep it in the field of view by autofocusing, and preserve the continuity of motion appearance even at low frame rates. Keeping inter-frame target migration small allows the particle search space to be restricted, and the correspondence problem to be solved locally rather than globally. Active detection of time-dependent object contours prior to tracking improves performance with fluorescence microscopy images and does not limit the nature or shape of the object of interest (Manzo and Garcia-Parajo, 2015). This method is capable of detecting multiple objects, analyzing and quantifying the level of focus for each single visible object in the field of view, and generating viewpoint motions to optimize the target focus in real time.

We interleave the electrode axial penetration step with target position monitoring. Individual insertion steps are typically set to 3–4 μm in order to prevent the maximum target migration induced by a single step from exceeding half of the diameter of the smallest structures targeted (i.e., half of ≈5–8 μm). The actual target migration distance in a step depends on the relative distance from the electrode tip and actual rheological property of the tissue (Cheng et al., 2008) (i.e., cortical layer, species, temperature, age, etc.). A typical approach trajectory, together with corresponding system parameters during approach, sealing, and break-in, is shown in Figure 2.

Our system is robust to breathing- and heartbeat-induced target motions, as they tend to induce the target to periodically disappear and reappear at the same point in the image. The interleaved step-and-reacquire method of our targeting strategy ensures that an electrode insertion step will not be executed until the center of mass coordinate of the target is updated and the electrode trajectory re-aligned. However, excessive breathing- and heartbeat-related movements may still be problematic for detection of object position during tracking, due to degradation of motion fluidity and apparent target deformation.

### Performance Evaluation

**Figure 3:**
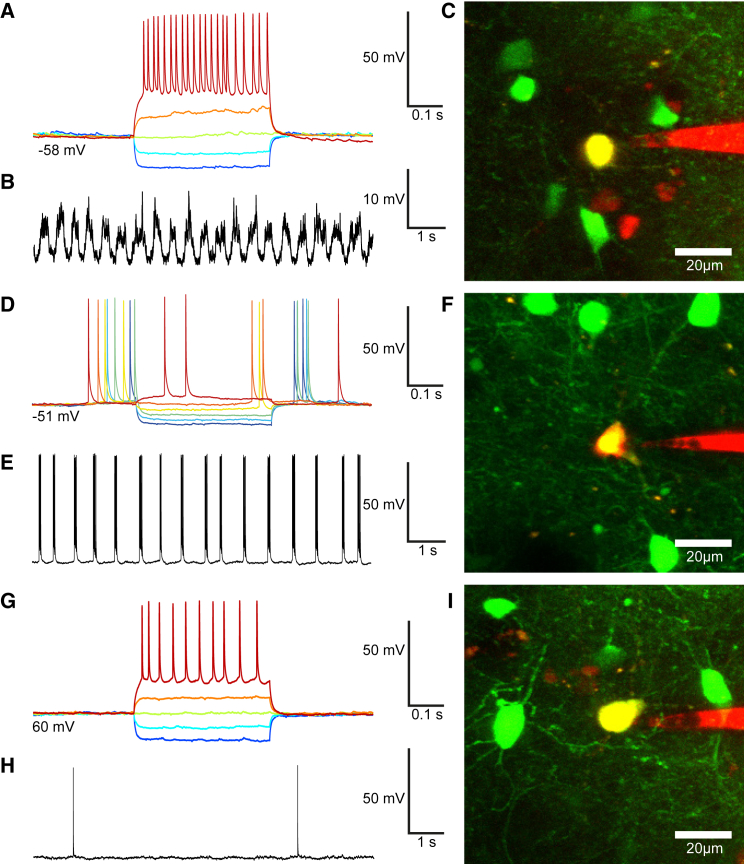
Further Examples of Robotic Two-Photon Targeted Whole-Cell Recording In Vivo (A and B) Current-clamp traces (A) during current injection (400 ms-long pulses from −100 to +100 pA in 50 pA steps) and (B) at rest for a patched neocortical interneuron in the V1 cortex of a GAD67-gfp (depth −113 μm from the brain surface; note compressed timescale relative to A). (C) Maximum-intensity z-projection of a two-photon stack image of the patched neuron and the electrode, acquired after the recording. (D and E) Current-clamp traces (D) during current injection (400 ms-long pulses from −100 to +25 pA in 25 pA steps) and (E) at rest for a patched neuron in the V1 cortex of a GAD67-gfp (depth −102 μm from the brain surface). (F) Maximum-intensity z-projection of a two-photon stack image of the patched neocortical interneuron and the electrode, acquired after the recording. (G and H) Current-clamp traces (G) during current injection (400 ms long pulses from −100 to +150 pA in 50 pA steps) and (H) at rest for a patched neuron in the V1 cortex of a GAD67-gfp (depth −142 μm from the brain surface). (I) Maximum-intensity z-projection of a two-photon stack image of the patched neocortical interneuron and the electrode, acquired after the recording.

We successfully used the platform to perform targeted whole-cell patch-clamp recordings from fluorescently labeled cells in a number of preparations, as shown in the example of Figures 1D–1G, which show current clamp traces obtained from a gfp-positive neocortical interneuron in a GAD67-gfp transgenic mouse. Further examples of successful recordings from neocortical interneurons are shown in Figures 3A–3I. We were also able to use GAD67-gfp to target cerebellar Purkinje cells and obtain cell-attached voltage-clamp recordings (Figure S2) and WCRs. The platform is not limited to the use of transgenic animals expressing fluorescent proteins, however: we were able to target selected pyramidal neurons in the primary visual cortex of wild-type mice by bulk loading with Oregon Green BAPTA-1 AM (Figures S3A–S3C), and were also successful in targeting astrocytes labeled with Sulforhodamine-101 (Figures S3D–S3F).

**Figure 4:**
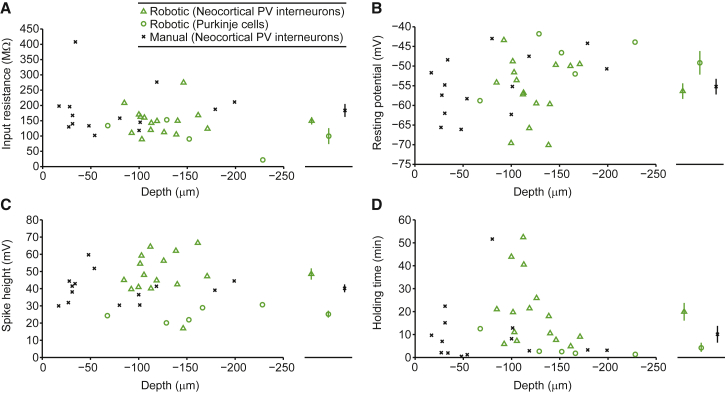
Visually Guided Robotic Patching Achieves Similar Results to Manual Targeted Patching Comparison of robotic and manual two-photon targeted recordings obtained from neocortical interneurons in the V1 cortex (triangles for robotic, n = 15; x for manual, n = 14) and from Purkinje cells in the cerebellum (circles for robotic; n = 5). Comparisons are shown as a plot of input resistances obtained versus cell depth (A; left) and mean access resistance ± SEM for each condition (A; right), resting potential versus cell depth (B; left) and mean resting potential ± SEM for each condition (B; right), spike height versus cell depth (C; left) and mean spike height ± SEM for each condition (C; right), and holding time versus cell depth (D; left) and mean holding time ± SEM for each condition (D; right).

The platform is capable of achieving gigaseal-tight contact between the patch pipette and cellular membrane, leading to WCR in either visually targeted or blind mode (in the latter case relying only on the pipette impedance signal; Figure S4). The quality and stability of the recordings obtained (quantified by input resistance, resting membrane potential, spike amplitude, and holding duration) are comparable to reports from both human operators (Margrie et al., 2002, DeWeese, 2007) and other robotic systems (Kodandaramaiah et al., 2012) across a range of recording depths (Figures 4A–4D and S4D). System performance is summarized in Table 1. A number of further performance characteristics can be appreciated. First, success rates in either robotic or manual two-photon targeted mode are lower than for robotic but blind recording. This simply relates to the fact that for “blind” recording, any cell is a potential target. Second, in addition to recording quality, the system achieves yields and operational speeds comparable to or exceeding human operators or other automated platforms for both whole-cell and cell-attached recording (the success rate for cell-attached recording is equivalent to the seal success rate in Table 1).

**Table 1:** System Performance for Robotic Two-Photon Guided Patch-Clamp Electrophysiology

	Robotic	n	Manual	n	Blind	n
Seal success rate (%)	46.6	90	56.4	78	74.2	35
WCR success rate (%)	22.2	90	18.0	78	51.4	35
Seals per animal	2.3 ± 0.3	18	4.9 ± 1.0	9	5.2 ± 0.3	5
WCR per animal	1.1 ± 0.2	18	1.6 ± 0.3	9	3.6 ± 0.4	5
Time to achieve seal (min)	6.0 ± 0.6	20	9.8 ± 1.4	14	3.3 ± 0.4	26
Time to achieve WCR (min)	6.1 ± 0.6	”	10.0 ± 1.4	”	3.6 ± 0.4	18
WCR holding duration (min)	16.6 ± 3.3	”	10.1 ± 3.6	”	–	–
Input resistance (MΩ)	139.2 ± 11.6	”	183.7 ± 21.0	”	64.1 ± 4.2	18
Series resistance (MΩ)	16.0 ± 0.5	”	16.3 ± 0.3	”	14.9 ± 0.5	”
Resting potential (mV)	−54.1 ± 1.8	”	−54.8 ± 2.0	”	−57.8 ± 4.8	”
Spike amplitude (mV)	42.7 ± 10.7	”	40.2 ± 2.3	”	50.1 ± 5.4	”

System performance for robotic two-photon guided WCR. Summary performance statistics for 90 patching attempts from 18 animals (1 craniotomy per animal) in robotically automated guidance mode, and 78 attempts from 9 animals (1 craniotomy per animal) in manual guidance mode. Animal age ranges were approximately matched (92 ± 39 versus 109 ± 25 days postnatal, mean ± SD). For comparison, the performance of our system in robotic but “blind” (non-visually targeted) is shown at right. Results are provided as mean ± SEM. n indicates the number of samples for the row statistic—number of patching attempts, number of animals, or number of successful recordings. Success rates in all cases exclude losses due to blockage of a pipette after insertion but before initiating cell approaching. Holding duration statistics for blind robotic recordings are not available; in these experiments, throughput was prioritized.

## Discussion

We have here reported a platform for fully robotic two-photon targeted WCR. Our technology platform, building on previous work (Kodandaramaiah et al., 2012), reduces the human variability, attentional load, and manual involvement of labor-intensive in vivo WCR experiments, lowering the entry barrier and leading to greater potential scalability. We have demonstrated an algorithm that circumvents the intrinsic constraints of two-photon imaging during tissue micromanipulation. Integration of a two-photon microscope with computer-controlled patch-clamp electrophysiology, together with the image processing method developed in this study, allows the selection of target cells based on genetic, morphological, and spatial information. One of the key technological challenges to solve was persistent monitoring of the target object position by autonomous computer vision, to assess and compensate for tissue movement induced by insertion of the pipette into the brain. Visual information is acquired from a time-lapse planar image stream by dynamically generating focal plane motion through autofocus methods while acquiring frames. The target is automatically kept in focus by an algorithm controlling the position of the objective lens based on the focus level of the target, and its position relative to the middle of the current field of view.

Pipette contrast is normally obtained by filling the pipette with a fluorescent dye such as Alexa. As the system keeps track of pipette location after initial acquisition, this dye can either be the same color as the target label, or a different color, although the latter is normally preferred. One constraint upon system performance can be the release of fluorescent dye from the pipette tip, accumulating in the tissue and increasing background fluorescence. This can compromise contrast and impact image quality, making computer vision and pipette re-acquisition difficult, especially after multiple insertions. An elegant solution to this problem would be to use quantum dot-coated glass pipettes (Andrásfalvy et al., 2014), which provide stronger two-photon contrast at greater depths, as well as eliminating dye ejection during entry.

The hit rate, recording quality, and time required for WCR using our method are comparable to that of a human operator performing manual two-photon targeted recording. In our testing, cell-attached recordings were obtained on 47% of attempts, and a successful whole-cell recording was achieved on 22% of occasions (i.e., just under half of the “seals” were converted to whole-cell recordings). Several (average 2.3) seals were achieved per animal, and just over one (1.1) whole-cell recording was obtained per animal. Yield was only slightly higher for manual targeted recording (4.9 and 1.6 seals and WCRs, respectively). In addition to fully robotic use, the platform can be used to accelerate manual two-photon targeted patching, and indeed hybrid manual/automated use may enable new recording capabilities such as in vivo targeted recording from fine dendritic processes. The platform is naturally extensible to parallel recordings from multiple micromanipulators/pipettes. As well as enabling the scaling up of two-photon targeted WCR, our system is likely to open up new application domains, via selective labeling of sub-cellular structures, and is likely to be applicable to more challenging experimental paradigms, such as targeted WCR of neurons in awake, behaving animals.

Combining the robotic two-photon targeted WCR system with functional imaging will enable numerous novel applications in neuroscience, extending our ability to study the molecular and cellular mechanisms of single-cell and network neuronal computation. Automated targeted electrophysiological interrogation of specific cells has the potential to provide not only readout of the intrinsic excitability, but also a direct means to determine the responsiveness of specific subtypes of neurons to different sensory stimuli, with high sampling throughput in comparison to sampling cells with “blind” approaches. Monitoring additional physiological parameters such as transient changes in intracellular calcium concentration or membrane voltage in neuronal populations will help to forge a direct link between system and cellular neuroscience. Another important application for this system might be assessing electrophysiological responses of individual cells transfected (and labeled) by recombinant viruses, plasmid DNA, or peptides suspected to modulate physiological functions. The increasing availability of genetically modified animals showing selective labeling in subpopulations of cells opens up numerous potential applications.

Further automation of procedures such as the fabrication (Pak et al., 2011), loading, and installation of fresh pipettes could eliminate time-limiting manual steps, enabling a single operator to run multiple experiments at the same time, either in the same or different animals. As well as enabling hitherto impracticable questions to be addressed concerning neural circuit function, this may be of great utility in testing pharmaceutical and gene therapeutic approaches to treating nervous system disorders.

## STAR★Methods

### Key Resources Table

**Table:** 

REAGENT or RESOURCE	SOURCE	IDENTIFIER
**Experimental Models: Organisms/Strains**		

Mouse: C57BL/6	Harlan UK and JAX	RRID: IMSR_JAX:000664
Mouse: GAD67-Gfp	Tamamaki et. al., 2003	GAD67-Gfp Mouse; MGI:3590301; RRID: IMSR_RBRC03666

**Software and Algorithms**		

Labview software	National Instrument	Labview 2012; RRID: SCR_014325
MATLAB software	MathWorks	MATLAB 2013a; RRID: SCR_001622
Sciscan software (ver 11 Dec 2014)	Scientifica	N/A
Software and relative dll file for third parties software interface	Axon Instruments	Multiclamp commander
Robotic Integrated Targeted Autopatcher (RITA) software	This paper	https://github.com/schultzlab/rita/

**Other**		

Two photon microscope	Scientifica	N/A
Galvanometric scanning mirrors	Cambridge Technology	6215H-671XX
Photo-multiplier tubes	Hamamatsu Photonics	R3896s
Dichroic beam splitter	Chroma Technology	T565LPXR
Optical filters	Chroma Technology	ET620/60M-2P
Optical filters	Chroma Technology	ET525/50M-2P
Mode-locked Ti-sapphire laser	Spectraphysics	Mai Tai HP
Water immersion objective, 0.8 N.A., 3.3 mmW.D.,	Olympus	LUMPLFLN-W 40 ×
Objective, plan achromat, 0.1 N.A., 18.5 mm W.D.	Olympus	RMS 4x
24 V / 65 W power supply	Maplin	N10NB
CCD Camera (based on a Sony CCD Progressive Scan Chip)	Scientifica	SciCam CCD Camera
Digital/analog acquisition board	National Instruments	DAQ PCI-6110
Digital/analog acquisition board	National Instruments	DAQ USB-6211
3axis micromanipulator	Scientifica	Multistar
Signal amplifier	Axon Instruments	Multiclamp 700B
Microcontroller (Arduino Due based on the Atmel SAM3X8E ARM Cortex-M3 CPU).	RS	Arduino Due
Custom assembled personal computer (Based on Microsoft Windows 7).	Scientifica	N/A
Pressure tap	Beefittings	FRLI 1/4, 2000 L/m
Flow control studs elbow	SMC Pneumatics	AS 2211F-01
Proportional solenoid valves	SMC Pneumatics	PVQ33-5G-16
Venturi vacuum ejector	SMC Pneumatics	ZH10DL-06-06-08
Flow control stud elbow	Beefittings	BSPT-PCSET 06-1/8
3 port solenoid valve	SMC Pneumatics	VQ110U-5M-Q
A Polytetrafluoroethylene (PTFE) tube (internal diameter 1.5 mm, wall thickness 0.3 mm)	VWE	DENE3401521
Bipolar NPN power transistors	ST Microelectronics	BD 237
NPN Darlington transistor	Digikey	TD - ZTX605
Diode	Transys Electronics	IN916
Pressure sensor	SMC Pneumatics	PSE 543-M3
Rectal probe and a feedback-controlled heating pad	FHC	40-90-8D
Dissecting microscope	Leica	S6E
Stereotaxic frame for small rodents	Harvard apparatus	724793
Dental drill	Osada Group	Success 40
Dumont Forceps – Dumostar size = 0.05 × 0.02 mm;	Fine Science Tools	11295-10
Ag/AgCl half-cell electrode 1.0 mm × 2.5 mm	World Precision Instruments	EP1
Manual pipette (0.5-10 μL)	Eppendorf	ES-10
Microloader tips (0.5 – 20 μL, 100 mm)	Eppendorf	Catalog No. 5242956003
Borosilicate capillaries with filament (76 mm, 1.5 mm outer diameter, 0.84 mm inner diameter)	World Precision Instrument	1B150F-3
Histoacrylamide	Braun Corporation	TS1050044FP
Dental acrylic	Kemdent	ACR811
Ophthalmic ointment	Allergan	Lacri-lube
Fentanyl (50 mg/ml)	Hameln pharmaceutical, UK	Sublimase
Midazolam (10 mg/ml)	Roche Products, UK	Hypnovel
Medetomidin (10 mg/ml)	Vetquinol, UK	Domitor
Alexa Fluor 594 hydrazide	Thermo Fisher and Molecular Probes	A10438

### Contact for Reagent and Resource Sharing

Further information and requests for resources and reagents should be directed to and will be fulfilled by the Lead Contact, Simon Schultz (s.schultz@imperial.ac.uk).

### Experimental Model and Subject Details

All procedures were carried out in accordance with institutional animal welfare guidelines and licensed by the UK Home Office under Project License 70/9095. Adult male and female mice (c57BL/6 wild-type mice, 8-12 weeks old, from Harlan UK, or GAD67-gfp, 2-5 months old), were housed in standard cages in the Imperial College London animal facility with ad libitum food and water in a controlled 12 hr light-dark cycle environment, with standard monitoring by veterinary staff.

### Method Details

#### Surgical Procedures

On the day of the experiment, the animals were anesthetized with a mixture of Fentanyl (Sublimase; 0.05 mg/kg), Midazolam (Hypnovel; 5 mg/kg) and Medetomidin (Domitor; 0.5 mg/kg) injected intraperitoneally and redosed at 90-120 min intervals with 50% of the initial dose as needed. Throughout all experiments, a rectal probe and a feedback-controlled heating pad maintained the animal temperature at 37 ± 1°C. A craniotomy (diameter 1.5 ± 0.5 mm) was made over visual cortex (1.5 mm anterior to lambda, 3.5 mm lateral from the midline) or over the cerebellum (1.0 mm posterior to lambda, midline) and a durotomy performed with forceps. Artificial cerebrospinal fluid (ACSF, consisting of 126 mM NaCl, 3 mM KCl, 1.25 mM NaH_2_PO_4_, 2 mM CaCl_2_, 2 mM MgSO_4_, 24 mM NaHCO_3_ and 10 mM glucose) was applied to keep the exposed cortex moist until pipette insertion. An incision in the middle upper part of the muscles over the neck was performed to firmly host an Ag/Cl grounded reference electrode (model EP1 Ag/AgCl half-cell electrode 1.0 mm diameter × 2.5 mm length; WPI).

#### Electrophysiology and Cellular Labeling

Whole-cell recordings were obtained upon establishing an electrical connection between a low-resistance (5 to 7 MΩ) micropipette (Sakmann and Neher, 1995) and an excitable cell membrane. We used heat-polished glass pipettes fabricated from filamented borosilicate capillaries (76 mm, 1.5 mm outer diameter, 0.84 mm inner diameter; code 1B150F-3 from WPI) pulled using a vertical pipette puller (model PC10, Narishige) and filled with an electrolytic solution. The internal solution was composed of 135 mM K-methanesulphonate, 7mM KCl, 10 mM HEPES, 2mM Mg-ATP, 2mM Na_2_-ATP, 0.5 mM Na_2_-GTP, and 0.05 mM ethylene glycol tetra-acetic acid (EGTA); the PH was 7.28 (measured with an Orion 9156BNWP combination pH electrode; Thermo Scientific); osmotic concentration was equal to 290 mmol/kg (measured with a vapor pressure osmometer model Vapro 5600 by Wescor Biomedical Systems). In some cases, two photon imaging contrast was due to endogenous labeling in GAD67-gfp transgenic mice. In other experiments, wild-type mice were fluorescently labeled with OGB-1 AM and SR-101, using previously established techniques (Stosiek et al., 2003, Nimmerjahn et al., 2004, Golshani and Portera-Cailliau, 2008, Schultz et al., 2009).

#### System design

The automated two-photon guided whole-cell recording apparatus is based on the integration of a commercial two-photon microscope (Scientifica) controlled by a data acquisition board (DAQ PCI-6110, National Instruments) and other devices used in a conventional in vivo electrophysiological setup, such as a 3-axis micromanipulator (Multistar patch clamp manipulator, Scientifica), a signal amplifier (Multiclamp 700B, Axon Instruments), a second data acquisition board (DAQ USB-6211, National Instruments) for electrophysiological data acquisition, and a personal computer running Microsoft Windows 7. In addition to this, we incorporated a custom made pressure regulator system controlled by a commercial microcontroller (Arduino Due board based on the Atmel SAM3X8E ARM Cortex-M3 CPU).

Two-photon laser excitation is generated by a commercial mode-locked Ti-sapphire laser (Mai Tai HP) and scanned by two galvanometric scanning mirrors (6215H-671XX Cambridge Technology). Fluorescence signal collection is performed using two photo-multiplier tubes (R3896s, Hamamatsu Photonics), a dichroic beam splitter (T565LPXR, Chroma Technology) and two optical filters (ET620/60M-2P and ET525/50M-2P, Chroma Technology). Sciscan software (Scientifica) controls beam scanning and signal collection through a data acquisition board. Two-photon images of fluorescently labeled neuronal tissue are reconstructed and streamed, in real-time, to a dedicated custom software module responsible for object identification, target selection (via a point-and-click graphical user interface) and tracking. Tracking information is then used to control the manipulator and adjust the trajectory and dynamics of pipette navigation toward the target.

The manipulator is connected to the computer through a serial COM port and is controlled via a custom written library of functions based on National Instruments VISA drivers (National Instruments). Pipette electrode visualization in two-photon images is enabled by augmenting the internal solution with a fluorescent dye (e.g., Alexa Fluor 594 hydrazide, Molecular Probes). During the entire patch clamp attempt and until the beginning of recording session, this amplifier generates a square wave seal test signal (10 mV in amplitude at 10 Hz) used to continuously monitor pipette impedance. Seal test response and electrophysiological signals are amplified by a Multiclamp Amplifier (700B, Molecular Devices) and acquired by a second data acquisition board (DAQ USB-6211, National Instruments). The Multiclamp 700B amplifier is controlled through a software module based on a dynamic linking library (dll; by Axon Instruments). The electrode seal test signal is sampled by the DAQ USB-6211 board at 20 KHz and filtered using a low pass digital Bessel filter (10 KHz-cutoff frequency). The resistance is calculated as the ration of the applied voltage and the measured peak-to-peak current at each cycle of the test signal.

The custom made pressure regulator is controlled in closed-loop by a microcontroller running a proportional-integral-derivative controller (PID controller) algorithm. The PID controller regulates the current flowing through the valves of the system. The schematic of the design is reported in Figure S5. The input pressure is provided by a fixed pressure reservoir regulated by a wall mounted tap ([E1] - FRLI 1/4, 2000 L/m, Beefittings). Further pressure reduction is performed through two manually regulated flow control studs elbow ([E2, E3] AS 2211F-01, SMC Pneumatics). E2 and E3 constitute the positive pressure supply to two electrically regulated proportional solenoid valves ([A and B] PVQ33-5G-16, SMC Pneumatics) regulating the flow in the branch controlling positive and negative pressure in the pipette. The positive branch consists of the pneumatic series E3-A while the negative branch is E2-B-D. Component D is a Venturi vacuum ejector ([D] - ZH10DL-06-06-08, SMC Pneumatics). The positive branch includes another manually regulated flow control stud elbow ([E3] - BSPT-PCSET 06-1/8, Beefittings) serving as an exhaust port. The positive and negative branches are connected respectively to the normally open and normally closed input of a 3 port solenoid valve ([C] - VQ110U-5M-Q, SMC Pneumatics). All the pneumatic components of the system are mounted on a custom designed transparent poly-methyl-methacrylate (Plexiglas) platform. The levels of pressure used during pipette initial insertion or automatic navigation to a target are constant and pre-defined in the configuration of the system. However, the adaptive suction for seal enhancement procedure uses a more refined method which constantly monitor the pipette resistance, and depending on its value and rate of change dynamically alters the pressure SP of the PID controller, and reproduces the suction manoeuvers of human patch clamp operators. Suction is applied if the pipette impedance is failing to increase in excess of another threshold value $S_{\text{th}}$ over a time period $T_{\text{seal}}$ and its value is lower than 15% of the target seal resistance. The algorithm has a time limit to attain a seal or reflect a failure.

### Quantification and Statistical Analysis

Quantitative results describing system performance can to be found in Table 1, with further description in the caption thereof, and in Figure 4, with description in the caption thereof.

### Data and Software Availability

The LabView software component of the platform has been made available at https://www.github.com/schultzlab/rita.

## Author Contributions

L.A.A. and S.R.S. conceived and planned the system. L.A.A. designed and developed the system, designed and performed the experiments, and analyzed the data. A.R.M. performed the manual recordings. A.R.M., C.S.C., O.E.A., and P.C. provided input on system design, validation, and testing. L.A.A. and S.R.S. wrote the manuscript.

## Supplemental Information

Document S1. Figures S1–S5

Document S2. Article plus Supplemental Information
