# Supplementary material for: Robotic Automation of In Vivo Two-Photon Targeted Whole-Cell Patch-Clamp Electrophysiology
Source: Neuron. 2017 Aug 30;95(5):1048–1055.e3. doi: 10.1016/j.neuron.2017.08.018 (PMC5584670; doi:10.1016/j.neuron.2017.08.018)
Supplement: Document S1. Figures S1–S5 [file mmc1.pdf]

Neuron, Volume *95*

Supplemental Information

Robotic Automation of In Vivo Two-Photon

Targeted Whole-Cell Patch-Clamp Electrophysiology

Luca A. Annecchino, Alexander R. Morris, Caroline S. Copeland, Oshiorenoya E. Agabi, Paul Chadderton, and Simon R. Schultz

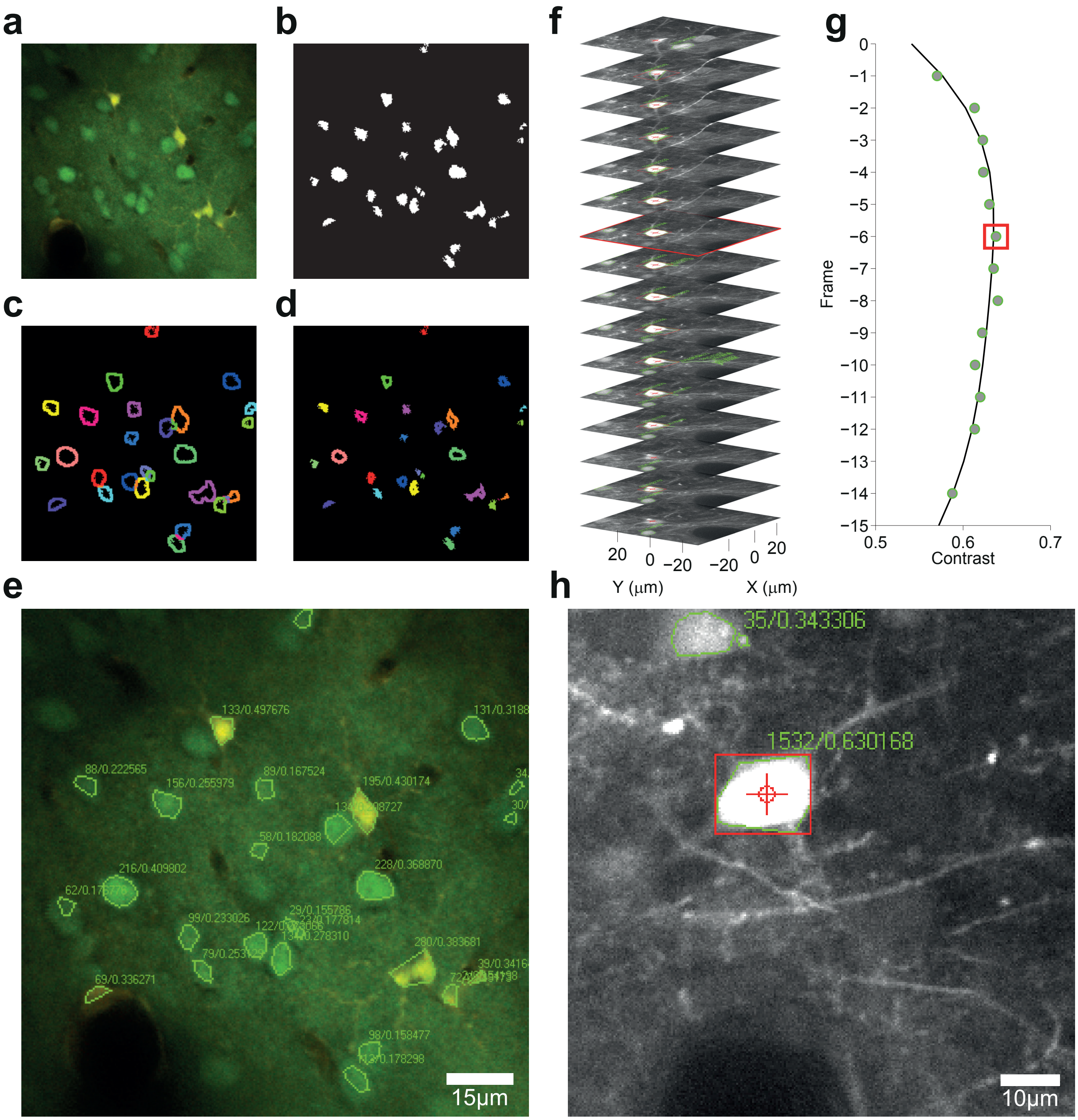

Figure S1. Related to Figure 1. Automatic analysis of two-photon images. (a) Initial image, an example of an RGB frame showing cortical tissue loaded with Oregon Green Bapta-1 AM and Sulforhodamine 101. (b) Erosion filtered version of the binary image obtained after thresholding the green channel (e) Set of masks used to select the external (c) and internal (d) boundaries of each segmented cell. (e) Analysis information is overlaid on the original image in real-time. (f) The focal plane is swiped over the evaluation volumetric region containing the target object and the contrast level of its light signature measured for each frame. (g) The plane with the best focus is identified by taking the maximum of a Gaussian function fitting the focus level of each plane. (h) At the end of the autofocus procedure the focal plane is set at the depth for which the maximum contrast was detected.

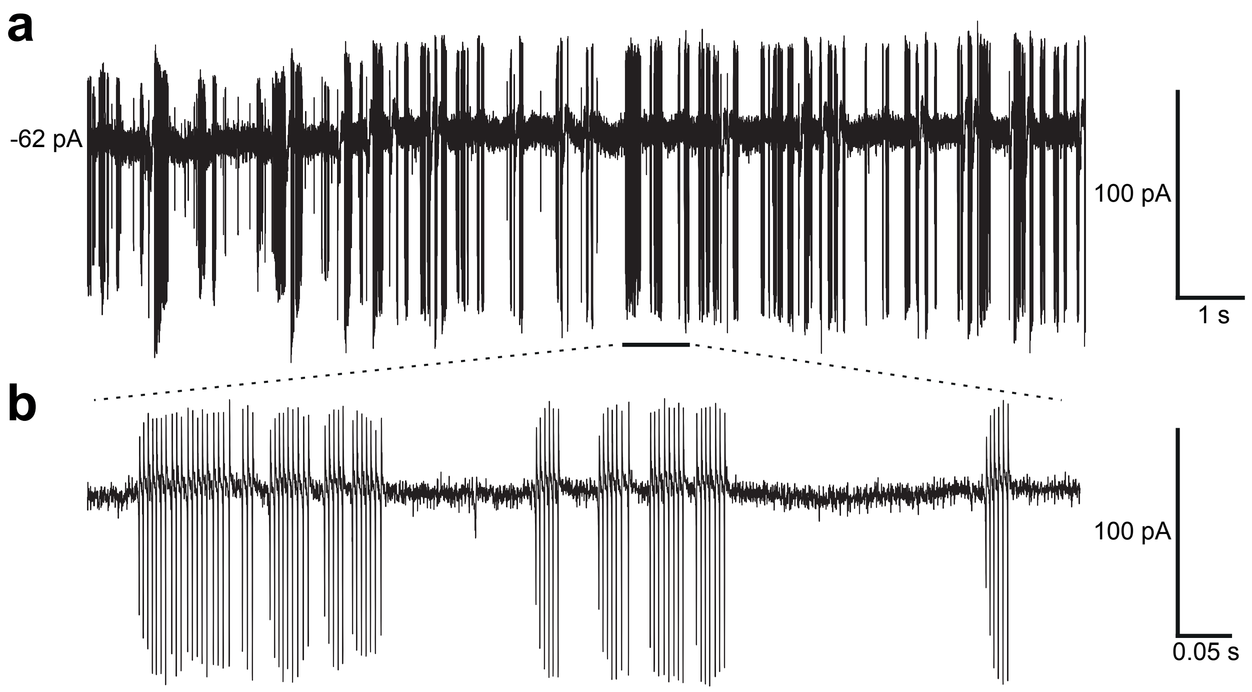

Figure S2. Related to Figure 3. Robotic cell-attached recordings. (a) Voltage clamp recording for a cerebellar Purkinje cell in a GAD67-gfp mouse, at a depth of 135 μm. (b) Expanded detail of the underscored section of the trace in (a).

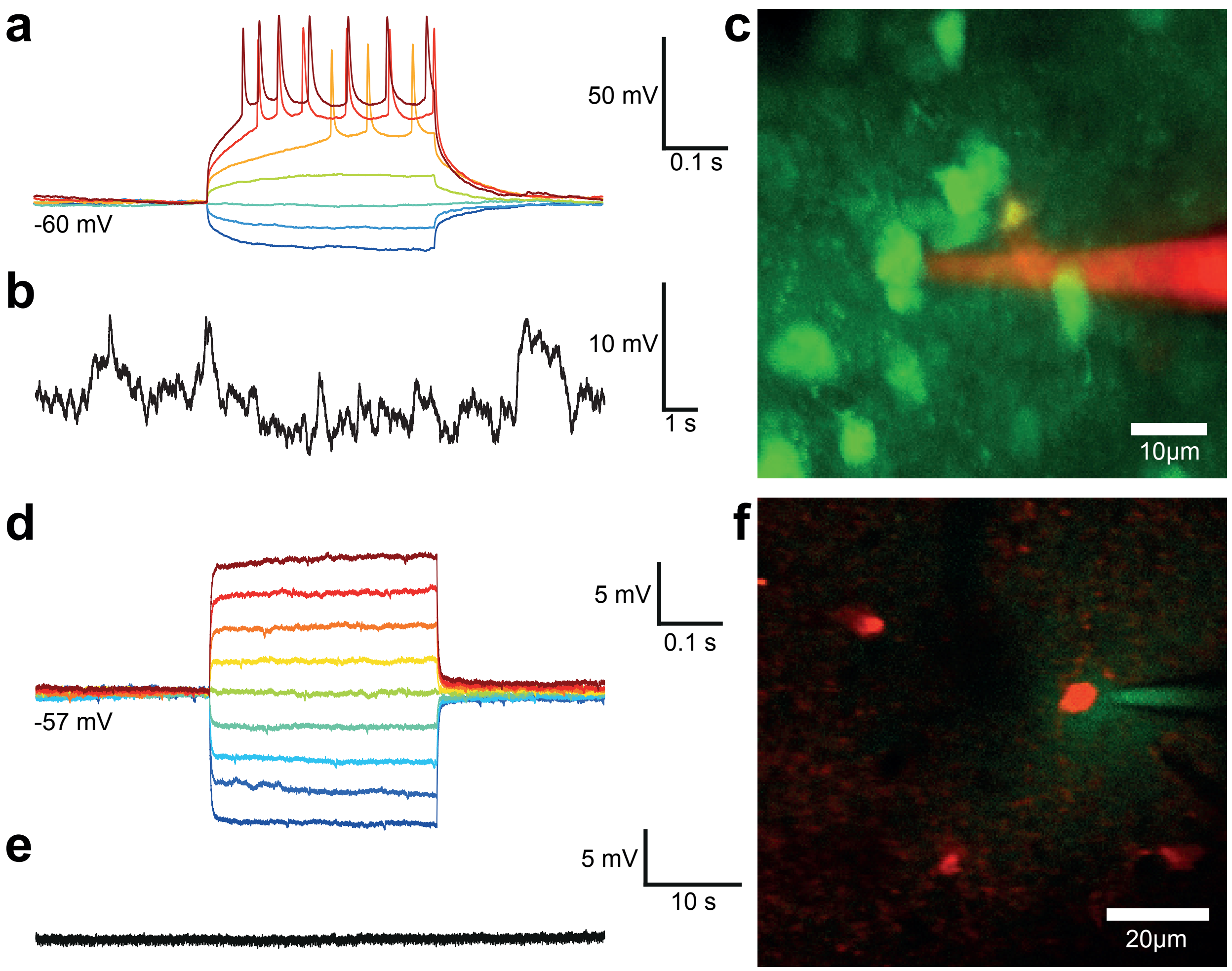

Figure S3. Related to Figure 3. (a) Current-clamp traces during current injection (400 ms long pulses from -50 to +100 pA in 25 pA steps) and (b) at rest for a patched neocortical neuron in the V1 cortex of a wild-type mouse bulk loaded with OGB-1 AM (depth -186 μm from the brain surface). (c) Maximum intensity z-projection of a two photon stack image of the patched neuron and the electrode, acquired after the recording. (d) Current-clamp traces during current injection (400 ms long pulses from -100 to +100 pA in 50 pA steps) and (e) at rest for a patched neocortical astrocyte in the V1 cortex of a wild-type mouse bulk loaded with Sulforhodamine 101 (depth -137 μm from the brain surface). (f) Two photon image of the patched astrocyte, acquired at the end of the targeting process.

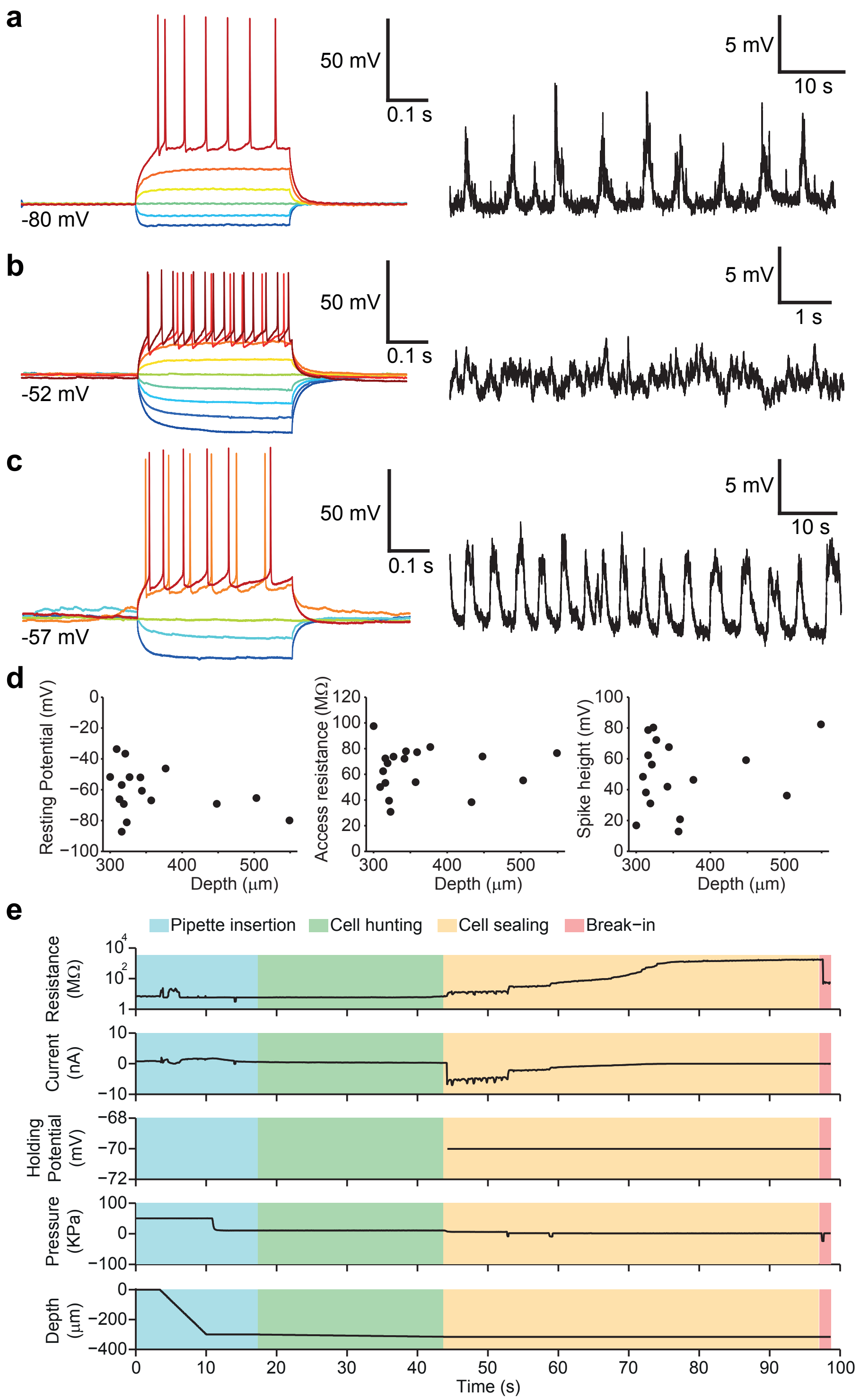

Figure S4. Related to Figure 3. Automatic blind whole cell recordings *in vivo*. (a; left) Intrinsic response of a neuron during hyperpolarising and depolarising current injection applied through the recording pipette electrode (400 ms-long current transients from -100 to +150 pA at 50 pA increase in steps); pipette tip depth was -549 μm from the pial surface. (a; right) Current clamp trace for the same neuron at rest. (b; left) Intrinsic

(Caption continued on following page)

response of a neuron during hyperpolarising and depolarising current injection applied trough the recording pipette electrode (400 ms-long current transients from -100 to 100 pA at 25 pA increase in steps); pipette tip depth was -342 μm from the pial surface. (b; right) Current clamp trace for the same neuron at rest. (c; left) Intrinsic response of a neuron during hyperpolarising and depolarising current injection applied trough the recording pipette electrode (400 ms-long current transients from -100 to 100 pA at 50 pA increase in steps); pipette tip depth was -316 μm from the pial surface. (c; right) Current clamp trace for the same neuron at rest. (f) Characterization of robotic blind recordings obtained from neurons in the V1 cortex (n=18) shown as a plot of resting potential versus cell depth, input resistances versus cell depth and spike height versus dell depth. (e) Example of time-courses of pipette resistance, current, holding potential, internal pressure and pipette depth. Time intervals relative to the different stages of the automated patching algorithm are colour coded. During the insertion process (in light blue) a relatively high internal pressure (50-100 KPa) was applied and the pipette was rapidly guided to a user defined depth. The pressure was then reduced to a relatively low level (5-10 KPa) and the hunting process (in green) initiated. A 15-20% increase in pipette resistance over three consecutive insertion steps indicates cell detection and initiates the sealing process (in yellow). Positive pressure was released and a holding potential of -70 mV applied to the pipette electrode. Adaptive suction was applied by the system to help attaining a tight seal. Cell break-in (in pink) was attained by applying one or more suction pulses. The cell patched at the end of this automatic patch clamp trials was a regular spiking neuron (intrinsic response shown in Fig.S4c).

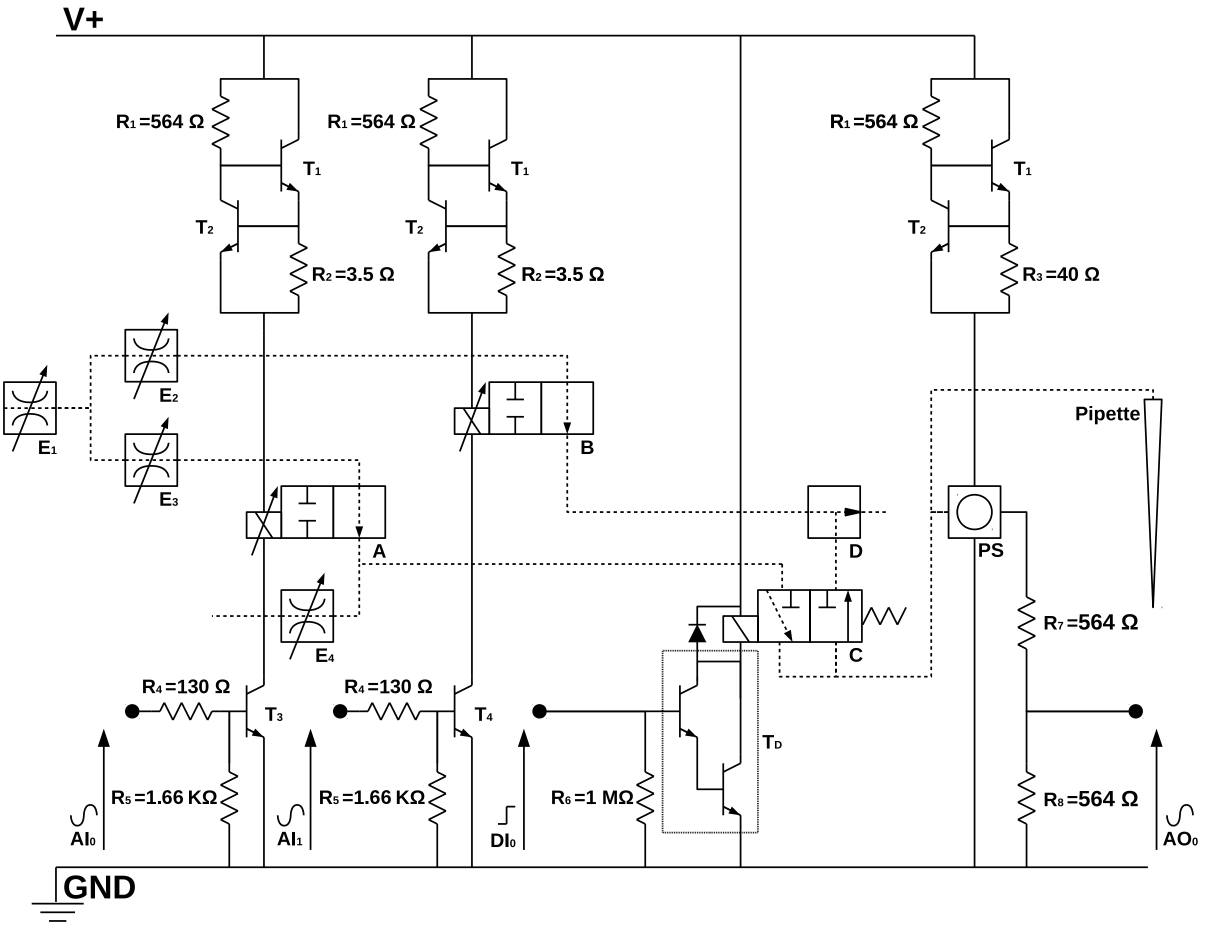

Figure S5. Related to Figure 1. Circuit schematics of the electro-pneumatic regulator. The electrical power supplied to A and B by the sub-circuits in the top left part of the schematics is regulated by two standard bipolar NPN power transistors (T_1 and T_2 - model BD 237, ST Microelectronics) controlled in the linear region (V_{be} = 0.5-1 V). The top left electrical sub-circuits use the same type of BJT and implement two current limiters. The 3-port solenoid valve (C) is an on/off type device controlled through a NPN Darlington transistor (TD - ZTX605, Digikey). A diode (IN916, Transys Electronics) is also included in the sub-circuit to protect this valve. The output port of C is then connected to the pipette and to a compound pressure sensor ([F] - PSE 543-M3, SMC Pneumatics) which provides the control system with the process variable values (1-5 V for -100 to +100 KPa). Terminal AO_0 in the schematic is connected to the terminal AI_0 of the microcontroller board. The proportional solenoidal valves A and B are controlled by means of two analogue signals generated by the 12-bit digital to analogue converter (DAC) on the microcontroller board. DAC output ranges from 0.58-2.8 V while the voltage range needed for the trans-conductance amplification is 0.5-1 V. Terminals AI_0 and AI_1 in the schematics are connected to the terminal AO_0 and AO_1 of the microcontroller. The control signals are in the range 0.5-1 V. Terminal DI_0 in the schematics is connected to the terminal DO_0 of the microcontroller.
